# Thinning effects on stand growth, carbon stocks, and soil properties in Brutia pine plantations

**DOI:** 10.1186/s13021-023-00226-0

**Published:** 2023-03-30

**Authors:** Neşat Erkan, Şükrü Teoman Güner, Ali Cem Aydın

**Affiliations:** 1 Faculty of Forestry, Bursa Technical University, 16310 Bursa, Turkey; 2 Department of Forestry, Ulus Vocational School, Bartın University, Ulus, 74600 Bartın, Turkey; 3 Southwest Anatolian Forest Research Institute, 07010 Antalya, Turkey

**Keywords:** Plant nutrient elements in soil, Silvicultural treatments, Carbon pools, Plant litter

## Abstract

**Background:**

The purpose of this study was to investigate the effects of thinning on stand growth, carbon (C) sequestration, and soil properties in Brutia pine (*Pinus brutia* Ten.) plantations. The study was conducted at two experimental sites -the Antalya-Kaş and Isparta-Eğirdir plantation areas- in Turkey between 1985 and 2015. Different thinning intensities -unthinned (control), moderate, and heavy- were replicated in four blocks. We determined the C in the living biomass, litter, soil, and some soil features for each experimental parcel.

**Results:**

We found no statistically significant difference in total stand volume between thinning-intensity treatments 30 years after thinning. This may be due to more light availability and less competition between trees and faster tree-diameter growth rate after thinning, thus explaining the volume in the treated parcels compared to the control over time. The C stocks in the biomass, litter, and soil were not significantly influenced by the thinning intensity. The nutrients in the litter and soil, and other soil properties, were not significantly different among thinning parcels. This implies that the C and other nutrients in the litter and soil are related to the stand volume and biomass, which were not changed by thinning in time.

**Conclusion:**

This finding is important in terms of showing that there was no change in total stand volume by thinning, which has been debated in the literature. This information is useful for forest managers when determining thinning strategy.

## Background

In addition to wood, forests provide many other goods and services, such as erosion control, carbon (C) sequestration, recreation, oxygen release, and aesthetic value. Thinning is one of the important silvicultural treatments used to maintain all these benefits. For instance, forests provide a useful means for the mitigation of global warming [1, 2], thus C sequestration is included in most forest management regimes. Therefore, efforts must be made to avoid impairing C and other nutrient turnover and soil fertility during the maintenance work involved in efficient forest management [3–5]. The practice of thinning -the removal of some of the trees from a stand as an intervention in forest growth- is very important in Mediterranean forests, where soil fertility is low and climatic conditions can be harsh [6–8].

Thinning improves light access in forest stands and speeds up litter decomposition by decreasing the density at the canopy level. It is commonly understood that thinning changes soil environments and the C and nutrient turnover rate in forest ecosystems [8, 9]. However, the amounts of C and nutrients and their turnover rates in forest stands is thought to be strongly related to biomass production [10–14]. To understand this better, this needs to be investigated, together with the effects of thinning on tree growth rates.

The main C stocks in forest ecosystems are the living tree biomass, dead organic matter and soil [Intergovernmental Panel on Climate Change (IPCC)], [15, 16]. Soil C and other nutrient stocks form a long-lived pool that requires studying over a period of time [2, 17]. Despite some studies having been published on the effects of thinning on forest C pools, few have been conducted on estimating the total C stock over the time, considering all forest repositories [18, 19]. Moreover, there have been conflicting results on the effects of thinning on wood production in forest stands. For instance, Hassenauer et al., [20] reported that, based on the literature, thinning generally does not increase total production at the end of the rotation period, although it can shift the distribution of growth to larger trees. However, Keyser and Zarnoch [21] reported that previous studies had shown a negative response of the aboveground live-tree C pool to thinning in the short and long term.

On the other hand, investigations of C stocks and nutrient sequestration at the tree species-specific level are essential for the reliable reporting of changes in C stocks. In this context, several tree species, including Mediterranean conifers, have been studied [7, 8]. However, although the effects of thinning on the total volume of Brutia pine (*Pinus brutia* Ten.) -a Mediterranean pine- have been investigated [22], there have been no studies published on the associated C sequestration. Brutia pine is a fast-growing tree species and has mean annual increment (MAI) more than 15 m^3^ha^−1^ at the age of 30 years in plantations [23]..

Brutia pine is an important tree species, not only because of its wide distribution in Turkey (5,736,371 ha, 25% of the total forested area), but also because of its wood, with 30% of Turkey’s industrial wood being produced in natural and plantation forests of Brutia pine [General Directorate of Forestry (GDF)], [24]. Furthermore, C sequestration in forest areas has been taken into consideration in management plans prepared by the Turkish GDF. Hence, the relationship between C sequestration in forests (in both the living biomass and the soil) and thinning as a silvicultural treatment needs to be studied in detail.

In this study, we aimed to reveal how different intensities of thinning affect the stand volume and C stocks in the living biomass, dead organic matter, and soil, as well as some soil properties, in Brutia pine forests. The results were expected to provide new scientific understandings to add to the knowledge base, while the findings will be useful for decision-makers in determining the most appropriate silvicultural strategy to be applied where C sequestration is combined with other objectives in management plans.

## Methods

### Study area

**Fig. 1:**
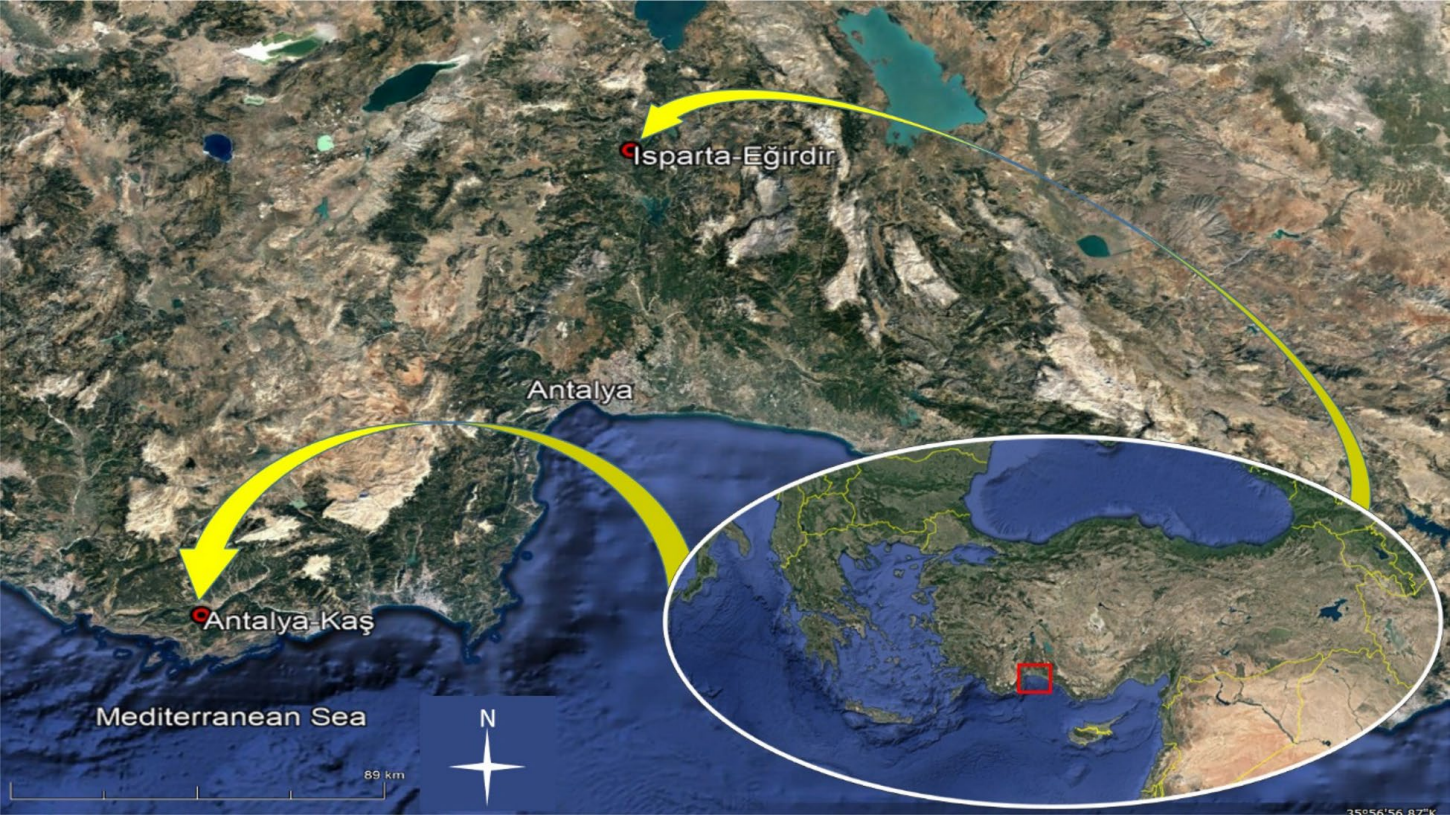
Locations of the experimental sites

The study area was located in the southwestern part of Turkey, and was conducted in four blocks at two sites -Antalya-Kaş-Tekircik and Isparta-Eğirdir-Aşağı Narlı- in Turkey both in Brutia pine plantations that were established between 1969 and 1973 (Fig. 1).

Research area has typical Mediterranean climate. Annual total precipitation for the Aşağı Narlı site is 1155 mm. The annual average, monthly average maximum and minimum temperatures were measured as 17.3, 24.7 and 12.1 ºC, respectively. These values were measured as 1200 mm and 17.9, 26.9 and 10.7 °C, respectively, for Tekircik site [25]. Meteorological data were calculated by interpolating the data of Karacaören (377 m asl.) and Kaş (5 m asl.) weather stations, which are the closest stations for Aşağı Narlı and Tekircik sites, respectively [24]. According to the Erinç method, cimate is humid in the both sites [26].

The Aşağı Narlı site is on conglomerate, while the Tekircik site is on limestone bedrock [27]. The Aşağı Narlı site consisted of sandy loam, sandy clay loam, sandy clay and clay loam; Tekircik site is located on silty clay loam, silty clay, clay loam and clay textured soils. The soils of both sites are unsalted and have alkaline reaction.

Typical woody taxa of Mediterranean vegetation, such as Brutia pine, kermes oak (*Quercus coccifera* L.), olive (*Olea europaea* L.), sandalwood (*Arbutus andrachne* L.), big nut (*Arbutus unedo* L.).), myrtle (*Myrtus communis* L.), laurel (*Laurus nobilis* L.), carob (*Ceratonia siliqua* L.), rosary (*Styrax officinalis* L.), redbud (*Cercis siliquastrum* L.) and oleander (*Nerium oleander* L.) types have been identified in both sites. Table 1 provides some of the other properties of the sites.

**Table 1:** Some properties of the experimental sites

Site	Block No	Parcel No	Site index (t = 35)*	Treatment	Elevation (m)	Coordinates	Stand age**
Kaş (Tekircik)	I	1	15.5	C	225	35 S 743,935 UTM 4,017,291	51
		2	15.1	H			
		3	14.8	M			
	II	1	11.5	H	245	35 S 742,867 UTM 4,014,843	50
		2	11.2	C			
		3	11.0	M			
	III	1	117	C	325	35 S 743,771 UTM 4,017,267	47
		2	12.2	H			
		3	12.1	M			
Eğirdir (Aşağı Narlı)	IV	1	12.9	H	395	36 S 0,301,707 UTM 4,162,770	49
		2	13.0	C			
		3	13.8	M			

^*^Site index is the top height at the age of 35

**stand age is the age in measurement year of 2015, letters in the “Treatment” column represent thinning intensity as *C* control, *H* heavy, and *M* moderate

### Experimental design and sampling procedure

The experiment was established through a randomized block design in 1985 and three treatments -a control, and heavy and moderate selective thinning- were performed in each block. Each treatment was represented by a parcel sized of 0.1 ha (33.3 × 33.3 m). Thinning intensity is generally quantified as the proportion of the total basal area of removed trees to the total basal area of the stand [20, 28]. We applied thinning in two intensities, cutting 35–40 and 15–20% of the basal areas, which can be described as “heavy” and “moderate” in thinning below [29, 30], respectively; there was no thinning in the control parcels. The post-thinning residues were harvested and removed from the forest. Thinning was repeated successively in 1990 and 2005 to maintain the initial density. Measurements of stand growth, based on individual tree diameter at breast height (dbh) and height, were performed in 2015. Total stand volume for different level of thinning intensities was calculated by summing of removed volume with final stand volume. Litter sampling was carried out at three different points in each parcel using a 25 × 25 cm frame. Then, a single sample was obtained for each parcel by mixing these three samples (i.e., 4 blocks × 3 thinning intensities × 3 replications = 36 litter samples). Soil samples were taken from the same points the litter samples were taken from in each parcel using a 1,000-cm^3^ cylinder, at three depth intervals 0–10, 10–20, and 20–30 cm (i.e., 4 blocks × 3 thinning intensities × 3 replications × 3 depth intervals = 108 soil samples).

### Laboratory analysis

The soil samples were air-dried, weighed, and sieved using a 2-mm-mesh screen. The dry bulk density was determined using the core method [31]. The soil texture was determined using the hydrometer method [32]. We determined the moisture in the soil samples based on drying at 105 °C. The organic C was determined using the Walkley–Black wet-oxidation method [33]. The pH was determined using an electrometric method in a soil/water solution in a ratio of 1:5 (v/v) [34]. The electrometric method was used to determine the electrical conductivity (EC) in a 1:5 (m/v) solution of soil/water [35], and the total calcium carbonate (CaCO_3_) was determined using a Scheibler calcimeter [36]. We determined nitrogen (N) using a modified Kjeldahl method [37], phosphorus (P) using a modified Bray and Kurtz No. 1 method, potassium (K) [33], Ca and magnesium (Mg) by the ammonium acetate method [38], and sulphur (S) by the turbidity method [39].

The litter samples were dried at 65 °C for 24 h before being weighed and ground up. A LECO CNH TruSpec elemental analyzer (Leco Corporation, St. Joseph, MI, USA) was used for the C analysis. The N was determined using a modified Kjeldahl method employing a FOSS 8400 automatic distillation unit [37]. Wet-ashed samples in nitric-perchloric acid were used to measure the P and S in a Shimadzu UV-1800 spectrophotometer using vanadomolybdophosphoric yellow color and turbidimetric methods, respectively. A Shimadzu 6601-F atomic absorption spectrometer was used to measure the Ca and Mg, and a Jenway PFP 7 flame photometer to measure the K [40].

### Data analysis

The stem volume of the trees in the experimental sites was determined using Eq. 1, based on the dbh and height, as developed by [23] for Brutia pine plantations. An adjustment factor (df = 1.007987) was used for the logarithm.

1 $$ln\, V=\mathrm{ln}\, (-2.0775)+ 1.6768\,\mathrm{ ln }\,d+ 0.8451\,\mathrm{ln}\,h$$

where *V* = tree volume (dm^3^), *d* = dbh (cm), and *h* = tree height (m).

The C stocks in forest ecosystems comprise: i) the above- and belowground biomass; ii) dead organic matter (i.e., deadwood and litter); and iii) soil organic matter [15, 16] (Eq. 2). We calculated the C sequestration by thinning intensity (treatment) in the biomass (aboveground + belowground) using Eq. (3). The total stem volume in the thinned parcels was obtained by adding the biomass removed via thinning for each related treatment to the final stand volume in the measurement year. For the control parcels, naturally eliminated trees were included in the total volume calculation. The C in the litter and soil was calculated using Eqs. (4) and (5), respectively. The soil depth for the C calculations was taken as 30 cm. The total biomass was estimated using Eq. (6).

2 $$Total \, C\, = \,C_{Biomass} \, + \,C_{Litter} \, + \,C_{Soil}$$

3 $$C_{Biomass} \, = \,V \, * \, D \, * \, BEF1 \, * \, (1\, + \,R) \, * \, CF_{B}$$

4 $$C_{Litter} \, = \,TL*CF_{L}$$

5 $$C_{Soil} \, = \,TS* \, CF_{S}$$

6 $$Total \, Biomass\, = \,\,V \, * \, BEF1 \, * \, (1\, + \,R),$$

where *C*_*Biomass*_ = C stocks in the current biomass (t C ha^−1^); *C*_*Litter*_ = C stocks in the litter (t C ha^−1^); *C*_*Soil*_ = C stocks in the soil (t C ha^−1^); *V* = stem volume (m^3^ ha^−1^); *D* = basic wood density (t m^−3^); *BEF1* = biomass expansion factor for converting the stem volume (including bark) into aboveground biomass (dimensionless); *R* = root:shoot ratio (dimensionless); *CF*_*B*_ = C fraction in the dry biomass [t C (tons dry biomass)^−1^]; *TL* = total litter (t ha^−1^); *CF*_*L*_ = C fraction in dry litter [t C (tons dry litter)^−1^]; *TS* = total soil (t ha^−1^); and *CF*_*S*_ = C fraction in soil [t C (tons soil)^−1^].

We used a biomass expansion factor (BEF1) of 1.2209 for the aboveground biomass, as given by Sun et al. [41] and a basic wood density (*D*) of 0.478, as calculated by As et al. (2001) for Brutia pine. The root:shoot ratio used was 0.229, as calculated by Ruiz-Peinado et al. [42] for *Pinus halepensis*, which is biologically very close to Brutia pine, and for the C fraction in the dry biomass (*CF*_*B*_), we used 0.51, as provided by the Land Use, Land-Use Change, and Forestry and Agriculture, Forestry, and Other Land Use guidelines for Turkish conifers [16].

An analysis of variance (ANOVA) was performed to test for differences in the C sequestration, biomass, litter, and soil from the different thinning intensities. Duncan tests were performed for multiple comparisons of the means that were found to be statistically significant in the analyses. For the statistical analyses, we used SPSS software [43]. Soil features, such as C and nutrient concentrations, were taken as dependent variables to investigate the effects of thinning on the accumulation of C and nutrients in the soil and biomass.

## Results and discussion

### Effects of thinning on stand growth, and soil and litter properties

#### Stand growth

The results obtained in the 2015, 30 years after treatment, showed that the number of living trees in the control parcels was significantly higher than in the treatment parcels. The number of remaining trees in the control parcels also decreased in time, during the study period due to competition among the trees, but the number still remained higher than in the treatment parcels. Therefore, the decrease in stocking in the control parcels by natural process was relatively slower than in the treatment parcels. The number of trees differed between parcels thinned moderately and heavily. However, this difference was not statistically significant (Table 2), possibly due to the high variance of the measured data. These results are generally compatible with those of a previous study by Kalıpsız [28].

**Table 2:** Stand parameters measured 30 years after treatment (in 2015) and their statistics by thinning intensity (mean ± SE)

Stand parameter	Thinning intensity		
	Control	Moderate	Heavy
Number of trees	2205 ± 315a	1172 ± 117b	1005 ± 205b
Mean diameter (cm)	18.09 ± 1.85a	20.54 ± 1.00b	21.94 ± 1.21b
Mean height (m)	16.54 ± 1.21a	18.24 ± 0.88b	18.30 ± 0.85b
Total basal areas (m^2^ ha^−1^)	53.76 ± 7.60a	39.90 ± 4.08a	44.89 ± 4.09a
Total stand volume (m^3^ ha^−1^)*	372.51 ± 57.36a	326.59 ± 37.82a	328.63 ± 29.41a
Total biomass (t ha^−1^)**	296.25 ± 45.62a	259.73 ± 30.07a	261.34 ± 23.39a

Significant differences among thinning intensity are indicated by a, b for stand parameters (*P* > *0.05*)

*Removal volume is included in total stand volume

**Total biomass is oven dry biomass of above + below ground biomass of trees

We observed a difference in mean stand diameter based on the thinning intensity. Indeed, diameter was one of the stand parameters most affected by neighborhood relations. For instance, Varmola and Salminen [44] reported a difference in mean dbh by thinning intensity after a period of 23–25 years in *Pinus sylvestris* stands. Similarly, Reukema and Bruce [45] determined a mean dbh of trees cut at the final harvest in thinned Douglas fir stands that was 5–35% larger than that of unthinned stands. Other studies have shown the significant effect of thinning on dbh [7, 28, 46–48]. In contrast to dbh, some previous studies have indicated that stand height increment and mean dominant height are not influenced by thinning [8, 48–50]. However, we calculated a significantly higher mean stand height for the thinned stands compared with the control stands (Table 2). This may be due to the fact that the trees removed by thinning were mostly smaller than the overall stand average (thinning below) and so a mechanical increase was observed in the mean height of the residual stand.

**Fig. 2:**
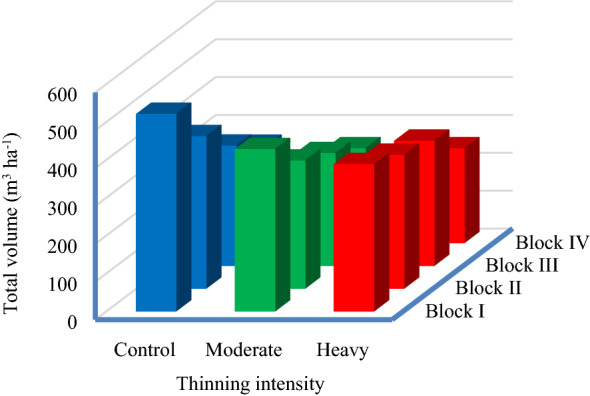
Total volume by thinning intensity 30 years (trees 42–46 years old) after treatment

We found no statistically significant difference in total volume between the treatments 30 years after thinning (Table 2, Fig. 2). This may be the result of a faster growth rate in diameter after thinning (age of thinning 12–16 years), which would affect the volume in the treated parcels compared to the control over time. Indeed, Brutia pine has been considered to be a fast-growing species with a mean volume increment of more than 10 m^3^ year^−1^ and with the mean volume increment reaching a maximum at 25–35 years, depending on the site index [23]. In addition, Brutia pine is a light-depended species [51], and after thinning, the trees receive more light and have less competition, which accelerates the growth of canopy and stem, and the increase in growth quickly compensate for the negative effect of removed trees on total volume in unit area.

Published findings by various researchers in this regard include, for instance, Hamilton [52] found no significant difference in the total volume of Norway spruce between four different thinning intensities after a period of 44 years. Similarly, Olume [50] determined that thinned and unthinned stands produced the same total volume of gross annual growth for Douglas fir 35 years after commercial thinning. Additionally, Hasenauer et al. [20] reported that, based on Wiedemann [53], Assmann [54], and Pienaar [55], thinning generally did not increase total volume but, shifted the distribution of growth to larger, more highly valued trees.

On the other hand, some studies have reported the reducing effect of thinning on stand growth. For example, Cochran and Barrett [56] found that thinning to a spacing wider than 9.3 ft (2.83 m) significantly reduced growth in both the stand basal area and the cubic volume per acre 35 years after treatment in ponderosa pine stands. Similarly, Bravo-Oviedo et al. [57] reported that moderate thinning (average residual basal area of 65–79% that in the control plots) from below in Scots pine stands caused a significant reduction (28%) in stand biomass.

### Soil properties

No significant differences (*p* > *0.05)* were found in the bulk density, sand, silt, or clay fractions, pH, EC, total CaCO_3_, organic C, N, P, K, Ca, Mg, or S contents in the soil based on thinning intensity (Table 3). Similarly, Genç et al. (2012) found that thinning applied in black pine (*Pinus nigra* Arnold) stands made no significant difference to some soil properties, including pH, and the sand, silt, clay, pH, organic matter, N, P, K, Na, Ca, and Mg contents. Tufekcioglu et al. [58] also reported that thinning did not significantly affect the organic-matter content of the soil in young beech (*Fagus orientalis* Lipsky.) stands. In another study, conducted by Tolunay [59], selective thinning in young Scots pine stands did not significantly affect the organic-C content of the soil. However, in contrast to our findings, in a study carried out in oak [*Quercus petrea* (Matlusch) Lieb.] coppice forests, the soil organic-C and total-N contents, and pH at depths of 0–20, 20–40, and 40–60 cm were significantly (*p* < *0.001*) increased as a result of increased thinning intensity. This indication was explained by increased litter decomposition and biological activity, and the mixing of more dead herbaceous sap material (including the roots) with the soil due to the increased amount of light after thinning [60]. Likewise, in the study carried out to determine the effect of thinning on soil and litter properties in the short term in *Cunninghamia lanceolata* plantations, it was determined that soil organic matter, total nitrogen, available nitrogen, available potassium and available phosphorus density and pH value increased depending on the increase in thinning intensity [61]. In another study, conducted in Scots pine and spruce forests in northern and southern Sweden, it was reported that the C and N contents of the soil increased in all three areas subjected to different thinning severities 15–16 years after treatment [62]. In addition, Johnson et al. [63] found a significant change in the soil C for all thinning intensities in a broad-leaved mixed forest in the long term (16 years after treatment) and in pure *Pinus taeda* L. stands in the short term (5 years). In this study, the absence of any difference in terms of soil properties can be explained by the disappearance of the effect of thinning in the long term.

**Table 3:** Changes in soil properties resulting from thinning intensity (mean ± SE)

Soil properties	Soil depth (cm)								
	0–10 cm			10–20 cm			20–30 cm		
	Control	Moderate	Heavy	Control	Moderate	Heavy	Control	Moderate	Heavy
BD (g/l)	1016 ± 54a	1001 ± 52a	1013 ± 63a	1061 ± 57a	1073 ± 55a	1116 ± 67a	1074 ± 75a	1010 ± 47a	1067 ± 75a
Sand (%)	27.1 ± 3.7a	26.1 ± 3.8a	28.4 ± 5.2a	21.6 ± 4.1a	23.4 ± 3.5a	27.2 ± 5.9a	21.5 ± 3.6a	22.1 ± 3.4a	26.7 ± 6.5a
Silt (%)	28.7 ± 1.7a	27.9 ± 2.1a	29.4 ± 1.9a	30.1 ± 2.4a	29.8 ± 2.2a	29.9 ± 2.0a	30.5 ± 2.7a	31.8 ± 2.0a	29.6 ± 2.1a
Clay (%)	44.1 ± 2.8a	45.9 ± 3.1a	42.2 ± 3.6a	48.3 ± 3.2a	46.8 ± 2.6a	42.8 ± 4.3a	48.0 ± 3.0a	46.4 ± 1.7a	43.6 ± 4.9a
pH	7.6 ± 0.06a	7.7 ± 0.03a	7.7 ± 0.06a	7.7 ± 0.06a	7.8 ± 0.03a	7.8 ± 0.04a	7.7 ± 0.05a	7.8 ± 0.02a	7.8 ± 0.02a
EC (mS/cm)	0.21 ± 0.03a	0.23 ± 0.03a	0.21 ± 0.03a	0.21 ± 0.03a	0.22 ± 0.03a	0.21 ± 0.02a	0.22 ± 0.03a	0.21 ± 0.03a	0.19 ± 0.02a
TL (%)	14.8 ± 6.4a	10.6 ± 2.9a	15.1 ± 5.2a	16.1 ± 6.5a	17.2 ± 3.8a	21.0 ± 5.5a	21.4 ± 7.6a	21.3 ± 4.4a	23.6 ± 5.2a
OC (%)	4.55 ± 0.69a	4.02 ± 0.31a	3.74 ± 0.43a	2.93 ± 0.54a	2.48 ± 0.26a	2.58 ± 0.27a	2.15 ± 0.36a	1.99 ± 0.27a	1.97 ± 0.22a
N (%)	0.25 ± 0.02a	0.24 ± 0.01a	0.23 ± 0.02a	0.18 ± 0.02a	0.18 ± 0.01a	0.18 ± 0.02a	0.15 ± 0.02a	0.16 ± 0.01a	0.16 ± 0.01a
P (mg kg^−1^)	14.5 ± 2.0a	14.8 ± 1.5a	14.5 ± 1.7a	8.9 ± 1.6a	8.9 ± 1.5a	10.3 ± 1.3a	7.4 ± 1.6a	6.4 ± 1.1a	7.8 ± 1.1a
K (mg kg^−1^)	361 ± 19a	270 ± 58a	366 ± 31a	314 ± 22a	365 ± 46a	271 ± 32a	249 ± 20a	285 ± 33a	221 ± 33a
Ca (mg kg^−1^)	11,408 ± 90a	11,916 ± 67a	12,327 ± 68a	10,990 ± 65a	11,736 ± 64a	12,420 ± 61a	11,245 ± 74a	11,608 ± 61a	12,208 ± 69a
Mg (mg kg^−1^)	1062 ± 136a	1040 ± 92a	1026 ± 118a	916 ± 99a	940 ± 88a	918 ± 123a	854 ± 102a	824 ± 72a	896 ± 109a
S (mg kg^−1^)	5.55 ± 04.5a	5.65 ± 0.52a	5.13 ± 0.49a	4.19 ± 0.39a	4.38 ± 0.52a	3.90 ± 0.42a	3.92 ± 0.44a	3.15 ± 0.45a	2.84 ± 0.32a

*BD* bulk density, *EC* electrical conductivity, *TL* total lime (CaCO_3_), *OC* organic carbon

Significant differences among thinning intensity are indicated by a, b for soil properties (*P* > *0.05*)

### Litter properties

Significant differences (*p* < *0.05*) were found in terms of the litter K and S concentrations by thinning intensity, whereas the differences were not significant (*p* > *0.05*) in terms of litter mass, and the C, N, P, Ca, and Mg concentrations (Table 4). The K and S concentrations in the litter were found to be lower in the heavily thinned areas than the others. Likewise, Güner and Güner [64] reported no significant effect of thinning on the litter mass in *P. nigra* afforestation. Contrary to our results, Makineci [60] found decreased (*p* < *0.001*) total amounts of litter (leaf + fermentation + humus in kilograms per hectare) due to increased thinning intensity in a study conducted in oak coppice forests. In a study conducted by Cheng et al., it was determined that litter mass and total nitrogen density decreased depending on the increase in thinning intensity [61]. In another study, thinning applied at different intensities in Scots pine plantations did not affect the concentration of C in the litter [7]. In our study, the insignificant difference in litter mass between the thinning intensities can be explained by the fact that there was no difference in the total stem volumes in the treated plots. Therefore, in many studies, it has been reported that litter, and thus the litter mass, is directly related to the aboveground biomass [10, 37, 65].

**Table 4:** Change in litter properties by thinning intensity (mean ± SE)

Thinning intensity	LM (kg m^−2^)	C (%)	N (%)	P (mg kg^−1^)	K (mg kg^−1^)	Ca (mg kg^−1^)	Mg (mg kg^−1^)	S (mg kg^−1^)
Control	2.24 ± 0.17a	42.2 ± 1.29a	0.67 ± 0.03a	682 ± 15a	1617 ± 100a	30,416 ± 3151a	3161 ± 52a	897 ± 15a
Moderate	2.45 ± 0.39a	45.5 ± 0.90a	0.74 ± 0.03a	659 ± 16a	1502 ± 312^a,b^	22,371 ± 777a	3023 ± 338a	943 ± 11a
Heavy	3.32 ± 0.66a	45.6 ± 1.30a	0.71 ± 0.05a	693 ± 13a	1281 ± 67b	28,038 ± 3438a	2892 ± 121a	877 ± 8b
F	1.617	2.714	0.758	1.350	3.811	2.291	2.013	8.088
*P*	*p* > *0.05*	*p* > *0.05*	*p* > *0.05*	*p* > *0.05*	*p* < *0.05*	*p* > *0.05*	*p* > *0.05*	*p* < *0.05*

*LM* litter mass

Significant differences among thinning intensity are indicated by a, b for litter properties (*P* > *0.05*)

### Effects of thinning on carbon and nutrient stocks

#### Carbon stocks in ecosystems

**Fig. 3:**
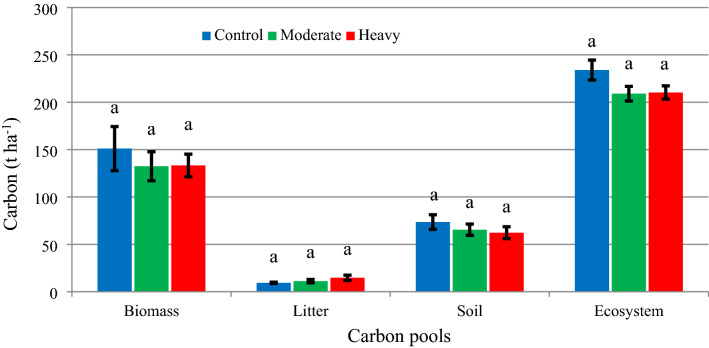
Comparison of C stocks by thinning intensity for different C pools (each column representing the mean value of the C stock for the related treatment). Error bars indicate ± SE The C stocks sharing the same letter in columns are not significantly different from each other at the α = 0.05 level, according to the ANOVA results for the biomass, litter, soil, and ecosystem

The C accumulated in the ecosystem was calculated as the sum of C in the biomass + litter + soil. The ANOVA results showed that the C stocks in the ecosystem did not differ with thinning intensity (Fig. 3). This may be due to the total biomass not being affected by thinning intensity in the experimental area. Therefore, the C sequestration and the turnover rate of the other plant nutrients in the forest ecosystem largely depended on the tree growth rate and, thus, the total basal area and volume stock in a given time period. Indeed, for example, litter and C sequestration in the forest ecosystem is calculated as a function of forest biomass [12, 13, 42, 66, 67]. Thus, the stand growth and total volume are important in terms of C calculations.

Similar to the ecosystem, the amounts of C in the biomass, litter, and soil, which are subcomponents of the ecosystem, were not affected by thinning intensity (Fig. 3), with calculations for the control, moderate, and heavy thinning intensities giving 151.1, 132.5, and 133.3 t C ha^−1^ in the biomass, 9.4, 11.2, and 14.7 t C ha^−1^ in the litter, and 73.5, 65.4, and 62.3 t C ha^−1^ in the soil, respectively (Fig. 3).

### Nutrient stocks in soil and litter

We found no significant differences (*p* > *0.05*), based on thinning intensity, in the C, N, P, K, Ca, Mg, and S stocks in the litter and soil (Fig. 2, Table 5). Likewise, in studies performed on Scots pine [68] and black pine [7], thinning applied at different intensities did not significantly affect the litter or soil C stocks (*p* > *0.05*). In another study, conducted by Cachinero-Vivar et al. [69], the effect of thinning on the soil C stock was found to be insignificant in Scots pine and black pine (*p* > *0.05*), whereas it *was* significant in maritime pine (*p* < *0.05*), with the highest C stock determined in moderately thinned plots. Güner & Güner [64] reported no significant effects of thinning on the litter C stock in black pine afforestation areas, but a significant effect on the soil C stock. In our study, the reason we found no significant difference in the litter and the C and nutrient stocks stored in the soil based on thinning intensity can be explained by the relevance of these amounts with tree biomass. Because an increase in below- and aboveground tree biomass led to litter and fine-root decomposition, the accumulation of organic C and nutrients in the soil increased. However, in our study, no significant difference was determined in either the C accumulation or the nutrient stocks because the stem volumes, and thus the biomass, in the treatment plots were no different, resulting in similar litter and fine-root decomposition.

**Table 5:** Change in nutrient stocks in litter and soil by thinning intensity (mean ± SE)

	Control	Moderate	Heavy	F	*p*
Litter					
N (kg ha^−1^)	154.41 ± 14.70a	186.06 ± 32.14a	251.4 ± 55.95a	1.675	> *0.05*
P (kg ha^−1^)	15.47 ± 1.46a	15.93 ± 2.36a	22.79 ± 4.36a	1.875	> *0.05*
K (kg ha^−1^)	37.19 ± 4.72a	36.03 ± 5.30a	44.42 ± 9.75a	0.426	> *0.05*
Ca (kg ha^−1^)	717.34 ± 120.73a	560.82 ± 100.39a	1148.77 ± 368.87a	1.713	> *0.05*
Mg (kg ha^−1^)	71.13 ± 6.13a	72.84 ± 10.49a	97.88 ± 20.12a	1.218	> *0.05*
S (kg ha^−1^)	20.04 ± 1.51a	23.26 ± 3.87a	29.38 ± 5.94a	1.283	> *0.05*
Soil (Ø < 2 mm)					
Total lime (t ha^−1^)	470.82 ± 202.55a	402.90 ± 89.50a	422.70 ± 116.69a	0.058	> *0.05*
N (kg ha^−1^)	4782 ± 431.60a	4610.25 ± 460.62a	4493.00 ± 504.06a	0.097	> *0.05*
P (kg ha^−1^)	24.00 ± 3.20a	22.91 ± 3.09a	23.66 ± 2.20a	0.037	> *0.05*
K (kg ha^−1^)	741.41 ± 49.30a	828.41 ± 73.14a	660.33 ± 77.76a	1.533	> *0.05*
Ca (t ha^−1^)	27.92 ± 2.81a	28.59 ± 3.14a	28.51 ± 1.65a	0.015	> *0.05*
Mg (kg ha^−1^)	2293.22 ± 279.01a	2258.75 ± 267.11a	2253.66 ± 335.39a	0.006	> *0.05*
S (kg ha^−1^)	10.91 ± 0.98a	10.33 ± 1.36a	9.16 ± 1.17a	0.564	> *0.05*

Significant differences among thinning intensity are indicated by a, b for nutrient stocks in litter and soil

## Conclusions

Brutia pine is one of the important tree species in the Mediterranean region, and covers 5.7 million ha of the forest area in Turkey. It is a fast-growing tree species that produces many benefits, mainly wood and C sequestration. In this sense, it is important to understand the effects of the alternative silvicultural regimes applied to Brutia pine forests, on stand volume, C sequestration, and soil properties for the better management of the other benefits. The results obtained from this study show that, thinning does not have a significant effect on the total stand volume, C sequestration, or litter and soil properties. These results suggest that management plans can be prepared in order to achieve other purposes in Brutia pine forests without having to consider thinning intensity as being deleterious. For instance, forest manager can apply the thinning regardless of intensity to shift the distribution of growth to larger, more highly valued trees and increase the overall economic value from plantation. In addition, our findings contribute to a better understanding of the effects of thinning on total volume, which has been a source of controversy in the literature.

## Data Availability

The datasets generated during and/or analyzed during the current study available from the corresponding author on reasonable request.

## Abbreviations

C: Carbon
IPCC: Intergovernmental Panel on Climate Change
GDF: General Directorate of Forestry
dbh: Tree diameter at breast height
EC: Electrical conductivity
N: Nitrogen
P: Phosphorus
K: Potassium
Ca: Calcium
M: Magnesium
S: Sulphur
t: Ton (Mg)
ha: Hectare
BEF1: Biomass expansion factor
D: Wood density
ANOVA: An analysis of variance
SE: Standard error
